# Computational exploration of two-dimensional silicon diarsenide and germanium arsenide for photovoltaic applications

**DOI:** 10.3762/bjnano.9.116

**Published:** 2018-04-19

**Authors:** Sri Kasi Matta, Chunmei Zhang, Yalong Jiao, Anthony O'Mullane, Aijun Du

**Affiliations:** 1 School of Chemistry, Physics and Mechanical Engineering, Queensland University of Technology, Garden Point Campus, QLD 4001, Brisbane, Australia

**Keywords:** density functional theory (DFT), photovoltaic applications, solar cell, two-dimensional semiconductors

## Abstract

The properties of bulk compounds required to be suitable for photovoltaic applications, such as excellent visible light absorption, favorable exciton formation, and charge separation are equally essential for two-dimensional (2D) materials. Here, we systematically study 2D group IV–V compounds such as SiAs_2_ and GeAs_2_ with regard to their structural, electronic and optical properties using density functional theory (DFT), hybrid functional and Bethe–Salpeter equation (BSE) approaches. We find that the exfoliation of single-layer SiAs_2_ and GeAs_2_ is highly feasible and in principle could be carried out experimentally by mechanical cleavage due to the dynamic stability of the compounds, which is inferred by analyzing their vibrational normal mode. SiAs_2_ and GeAs_2_ monolayers possess a bandgap of 1.91 and 1.64 eV, respectively, which is excellent for sunlight harvesting, while the exciton binding energy is found to be 0.25 and 0.14 eV, respectively. Furthermore, band-gap tuning is also possible by application of tensile strain. Our results highlight a new family of 2D materials with great potential for solar cell applications.

## Introduction

The potential applications of two-dimensional (2D) materials are one of the key research areas for many researchers since graphene was isolated and characterized in 2004 [1]. The number of applications is vast including photovoltaics, nanoelectronics, Dirac materials and solar fuels through water splitting, to name but a few. Although single elemental 2D materials from groups IV and V, such as like phosphorene and stanine, have been studied in detail [2–3], we focus our study on the less well-known combination of IV–V compound semiconductor materials at the two-dimensional scale for their possible electronic applications. The less extensively studied compounds SiAs_2_ and GeAs_2_ are considered here for a computational study. The synthesis of these compounds [4–5] was reported a few decades ago as well as their basic crystal structures and corresponding phase diagrams [6–7].

In 1963, Hulliger et al. [8] predicted that the group IV–V compounds SiAs, GeAs and GeAs_2_ would show semiconductor behavior. The synthesis and crystal structures of the group IV–V_2_-type compounds SiP_2_, SiAs_2_ and GeAs_2_ were reported later. All three compounds exhibit the orthorhombic space group *Pbam* with eight formula units per cell [4]. However, they did not report the band structure or the band gap values of these materials. Later, Wu et al. performed theoretical studies on silicon and germanium arsenides [9] to predict and reaffirm that m-SiAs/GeAs and o-SiAs_2_/GeAs_2_ are indeed semiconductors. The studies were based on band-structure calculations and are in agreement with experimental observations.

A recently reported computational study on 2D GeAs_2_ was performed to investigate its thermal conductivity and its suitability for thermoelectric applications [10]. In order to further study GeAs_2_ and to compare it with a similar material from the same IV–V group combination, we focus our study on two-dimensional SiAs_2_ and GeAs_2_ and compare them with their bulk counter parts with regard to electronic band structure, phonon-vibration frequencies, optical properties, band gap modulation behavior and predict their potential applications.

## Computational Details

Density functional theory (DFT) using plane wave Vienna ab initio simulation package (VASP) code is used for first-principle calculations for this study [11–12]. The geometry optimisation is done with generalized gradient approximation in the Perdew–Burke–Ernzerhof form (GGA-PBE) exchange–correlation functional [13]. A Monkhorst–Pack *k*-points grid [14] of 9 × 3 × 2 and 9 × 3 × 1 was used for sampling the first Brillouin zone for bulk and monolayer for geometry optimizations. Both bulk and monolayer systems are set for relaxing until residual force and energy converged to 0.005 eV/Å and 10^−7^eV, respectively. To study 2D monolayer systems under periodic boundary conditions, a vacuum layer of about 15 Å was introduced to minimize the spurious interaction between neighboring layers. The electronic band structure is predicted through hybrid density functional theory based on the Heyd–Scuseria–Ernzerhof (HSE) exchange–correlation functional [15–16] and Wannier90 package [17] implemented in the VASP code. The thermodynamic stability of the material is assessed by the formation energy for the 2D material and is indicated as “the difference in free energy of the 2D material and the lowest value of the bulk equivalent of the same material”, *E*_f_:

[1]
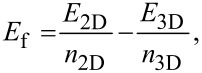


where, *E*_2D_ and *E*_3D_ are the energies of the monolayer and the bulk material, respectively. *n*_2D_ and *n*_3D_ are the number of atoms present in the unit cells considered for the calculations [18–20]. The optical properties are evaluated by determining the frequency-dependent dielectric tensor matrix through hybrid DFT based on the Heyd–Scuseria–Ernzerhof (HSE) exchange–correlation functional [15–16], implemented in VASP [21–23]. The corrected average electrostatic potential in the unit cell with regard to the vacuum potential is obtained from HSE band calculations. It was subsequently used as a generic reference for aligning the band positions that were obtained from HSE06-Wannier calculations. A zero-damped van der Waals correction was incorporated using the DFT-D3 method of Grimme’s scheme [24–25] to better describe non-covalent bonding interactions. The projector-augmented-wave (PAW) method [26] was used to describe the electron–ion interaction. Also, the plane-wave energy cut-off was set to ca. 255 eV and, in addition, a high precision option (PREC = high) is used in the input file, which would further set cut-off energy and other defaults such as grid spacing representing the augmentation charges, charge densities and potentials (NGFX, NGFY, NGFZ) so as to get accurate results. The phonon spectrum was computed using the density functional perturbation theory (DFPT) [27] as implemented in the Quantum-ESPRESSO package [28]. The excitonic properties are studied using Green’s function, GW-Bethe–Salpeter equation (BSE) approach implemented in VASP code [23,29–31]. The GW calculations were performed with a 13 × 5 × 1 *k*-grid, the energy cut-off for response function is set at 100 both in GW and BSE approach for exact compatibility. Over and above the GW results, the BSE method was adopted to obtain the light absorption spectrum [21–22] and the optical band gap. BSE was solved by using the ten highest valance bands and ten lowest conduction bands and with a 13 × 5 × 1 *k*-grid. Using the band gaps obtained from GW and BSE functional methods the exciton binding energy was obtained [32].

## Results and Discussion

**Figure 1:**
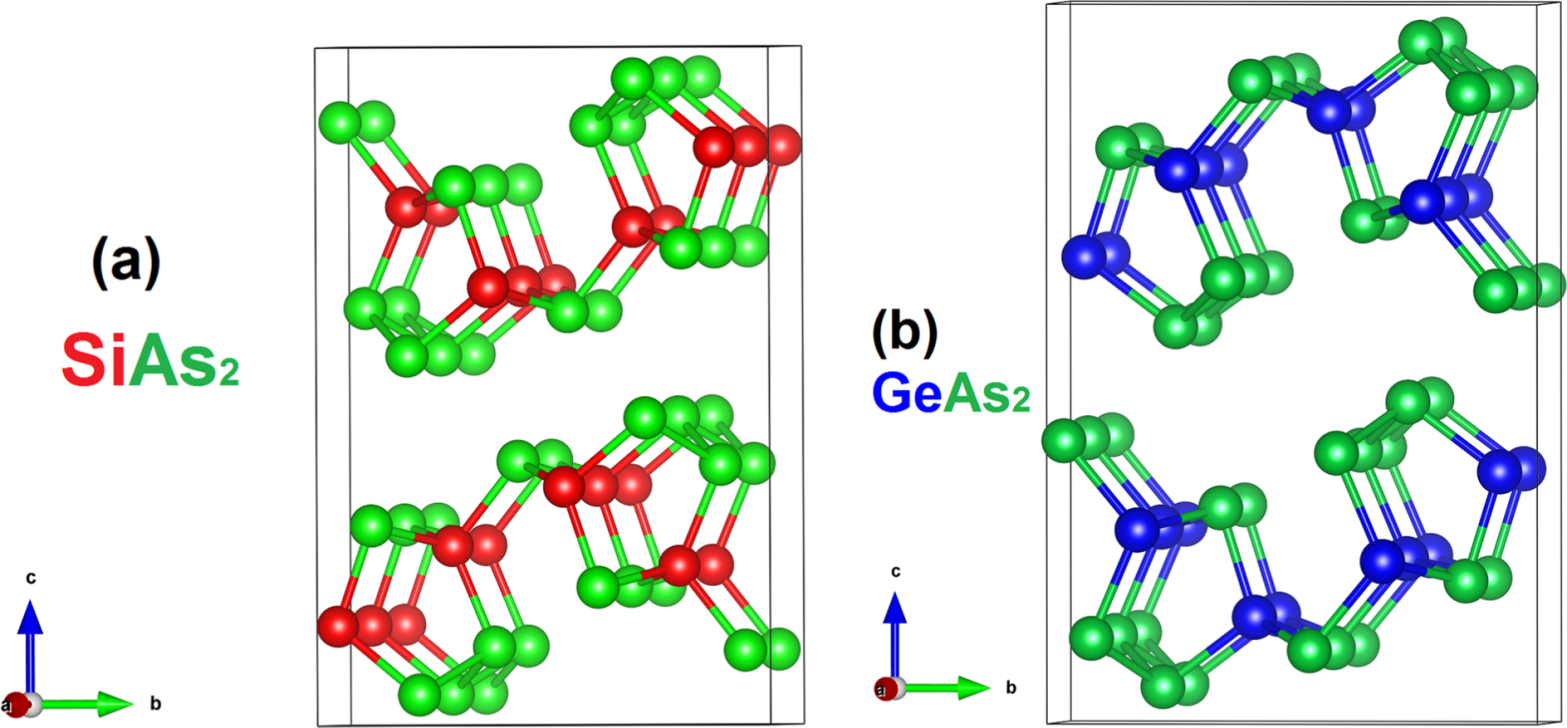
Crystal structure side view of (a) SiAs_2_ bulk (3 × 2 super cells) and (b) GeAs_2_ bulk (red: silicon, blue: germanium, green: arsenic).

The crystal structures of SiAs_2_ and GeAs_2_ are orthorhombic with the space group *Pbam* (no. 55) having eight symmetry operators or atoms in the unit cell [4]. Figure 1 shows the side views of the bulk configurations of SiAs_2_ and GeAs_2_ with atomic layers interacting with neighboring layers through weak van der Waals forces. Detailed structural parameters for bulk and monolayers of SiAs_2_ and GeAs_2_ are listed in Table 1. The calculated lattice constants for the bulk materials are in good agreement with previous experimental values [4]. The calculated lattice constants for single-layered SiAs_2_ and GeAs_2_ are slightly smaller than those calculated for the bulk phases. The lattice constant *c* is kept constant and greater than 15 Å by adding a vacuum layer.

**Table 1:** Calculated structural parameters of SiAs_2_ and GeAs_2_ compared with the experimental values.

	SiAs_2_			GeAs_2_		
	bulk (calc.)	bulk (exp.)	monolayer (calc.)	bulk (calc.)	bulk (exp.)	monolayer (calc.)

*a* (Å)	3.691	3.636 [4]	3.676	3.795	3.728 [33] (3.721 [34])	3.760
*b* (Å)	10.124	10.37 [4]	10.258	10.362	10.16 [33] (10.12 [34])	10.397
*c* (Å)	14.857	14.53 [4]	—	14.666	14.76 [33] (14.74 [34])	—

**Figure 2:**
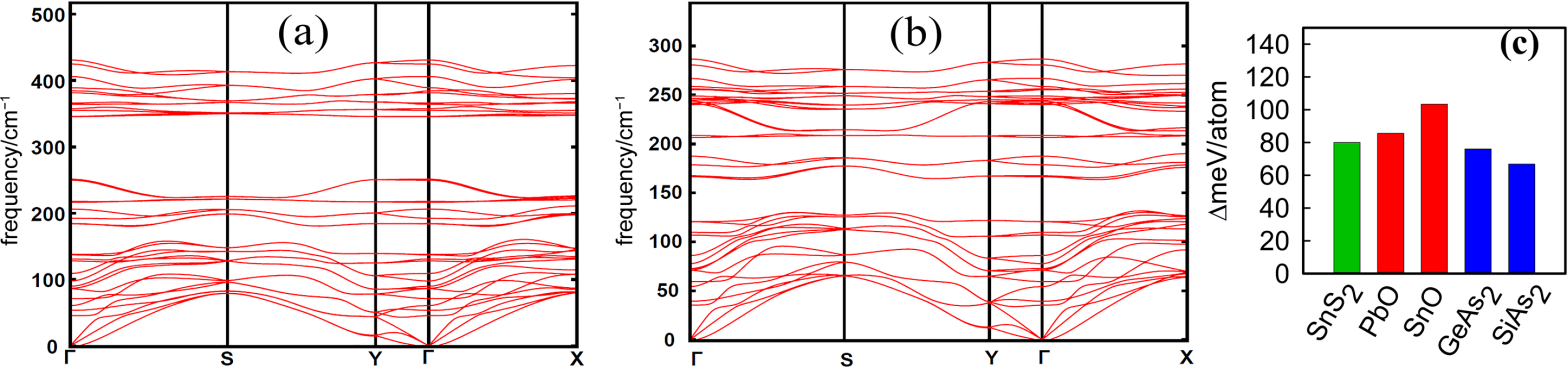
Phonon band structure of a monolayer of (a) SiAs_2_ and (b) GeAs_2_ along the high-symmetry points in the 1st Brillouin zone. (c) Energy of formation of the 2D material from its bulk counterparts (green: experimentally synthesized 2D compound, red: not experimentally synthesized 2D compound, blue: possibility to synthesize 2D compound theoretically shown).

The dynamical stability of SiAs_2_ and GeAs_2_ monolayers is evaluated by analysing the phonon band spectrum. As shown in Figure 2a and Figure 2b, no imaginary frequency can be found at any wave vector, confirming that the single-layer SiAs_2_ and GeAs_2_ are dynamically stable. The thermodynamic stability identified through the energy of formation, *E*_f_, calculated from Equation 1 determines the strength of van der Waals interactions in the bulk materials of SiAs_2_ and GeAs_2_. Thus, the lower the formation energy the easier it can be extracted from the bulk material. It has been reported that less than 200 meV/atom is considered to be a sufficiently low formation energy that the free-standing 2D layer can be extracted from the bulk material [20]. In our calculations, *E*_f_ for SiAs_2_ and GeAs_2_ is found to be 66.8 and 76.0 meV/atom, respectively. These values are smaller than the *E*_f_ values of already exfoliated 2D materials. The values are compared with the *E_f_* values reported for chalcogenides of Sn and Pb [20,35] and are depicted in Figure 2c. According to this evaluation, the exfoliation of monolayer for these materials from their bulk forms is highly feasible.

**Figure 3:**
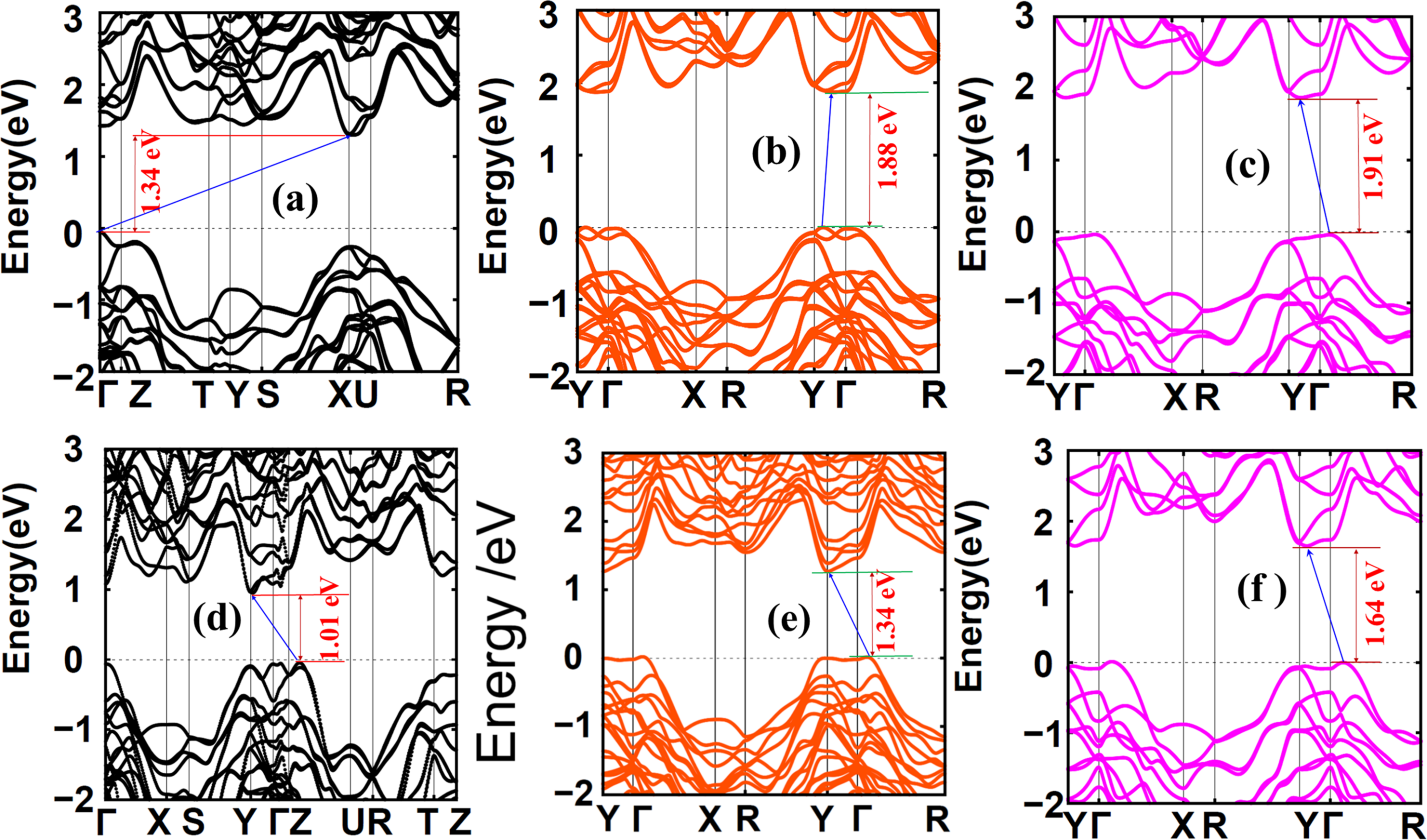
Band structure for SiAs_2_ and GeAs_2_ calculated by the HSE-Wannier function method. The Fermi level is set as zero. (a–c) Bulk, bilayer and monolayer of SiAs_2_, respectively; (d–f) bulk, bilayer and monolayer of GeAs_2_, respectively.

The band structures determined by HSE-Wannier calculations demonstrate that bulk, bilayer and monolayer structures of SiAs_2_ and GeAs_2_ (Figure 3a–d) are semiconductors with indirect band gaps (the VBM and CBM locations are marked). The bandgaps are given in Table 2. These results are consistent with previously reported calculated values for both bulk and monolayers of GeAs_2_ with values of 0.99 and 1.64 eV, respectively [10]. The decrease in the thickness of both SiAs_2_ and GeAs_2_ leads to a quantum confinement effect [36] and thus the band gap is increased significantly from the bulk to the monolayer structures in both materials. In addition, the experimentally reported indirect band gap of 1.06 eV by Rau et al. for single crystal orthorhombic GeAs_2_ is in agreement with our calculated result for the bulk material [5].

**Table 2:** Calculated band gaps of SiAs_2_ and GeAs_2_ for bulk, bilayers and monolayers.

	SiAs_2_			GeAs_2_		
	bulk	bilayer	monolayer	bulk	bilayer	monolayer

bandgap (eV)	1.34	1.86	1.91	0.99	1.34	1.64

**Figure 4:**
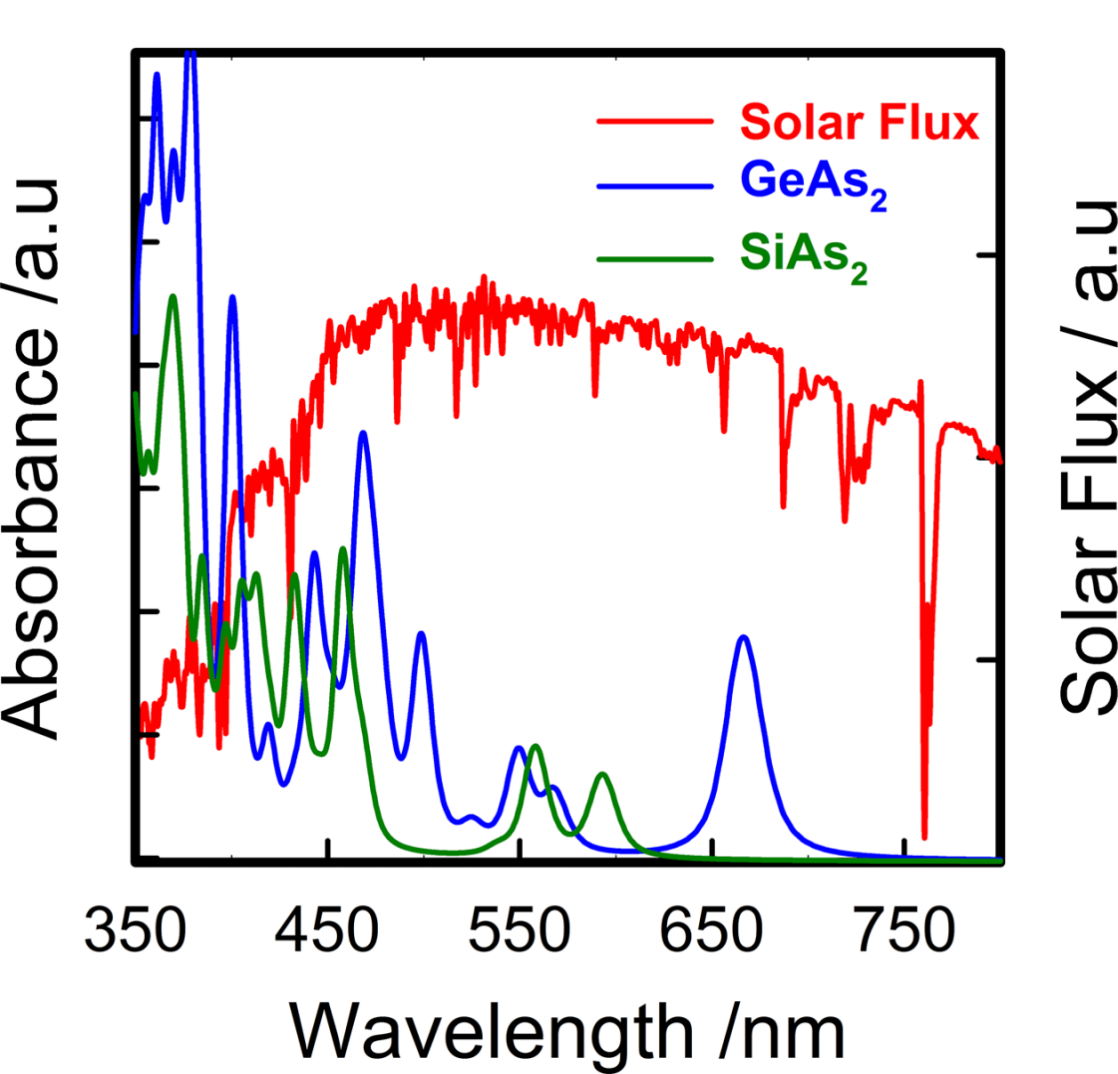
Calculated light absorption spectrum of monolayers of SiAs_2_ (green) and GeAs_2_ (blue) using HSE functional superimposed to the incident AM1.5G solar flux.

The ability of a GeAs_2_ monolayer to harvest solar light in the visible region is higher, both in absorption intensity and in the range of wavelengths covered, than that of SiAs_2_. This is shown in Figure 4, which shows the light absorption spectrum calculated from HSE functional in the visible light region (approx. 350–800 nm) compared with the AM1.5G solar spectrum. However, both materials exhibit an almost equal absorption in the wavelength region of 350–600 nm. Above this, GeAs_2_ still has good absorption up to around 700 nm.

It has been reported that exterior strain on semiconductor nanostructures, especially at the two-dimensional level, influences the electronic properties and the corresponding optical properties [37–38]. We, therefore, studied the PBE functional band gap variation as a function of tensile strain (Figure S1, Supporting Information File 1). The band gap variation depends on the viewing direction along the lattice. In the laboratory, an external strain can be imparted by different means such as adlayer–substrate lattice mismatch, external loading, bending or by applying stress on the material [39–42]. While the expansion or compressive strain in the direction of *a* can transform the semiconductor into a metal [18]. Higher strains (deduced by extrapolation, not shown in the figure) and compression can cause a continuous increase in the band gap from compressive strain to expansion strain in both SiAs_2_ and GeAs_2_. This indicates that there is the possibility of band gap tuning to make the semiconductor suitable for a desired electronic application. However, this band gap variation was calculated using a PBE functional that is known to underestimate the value of the band gap. However, for the purpose of the illustrating the dependence of the band gap variation on strain, these calculations can be helpful.

**Figure 5:**
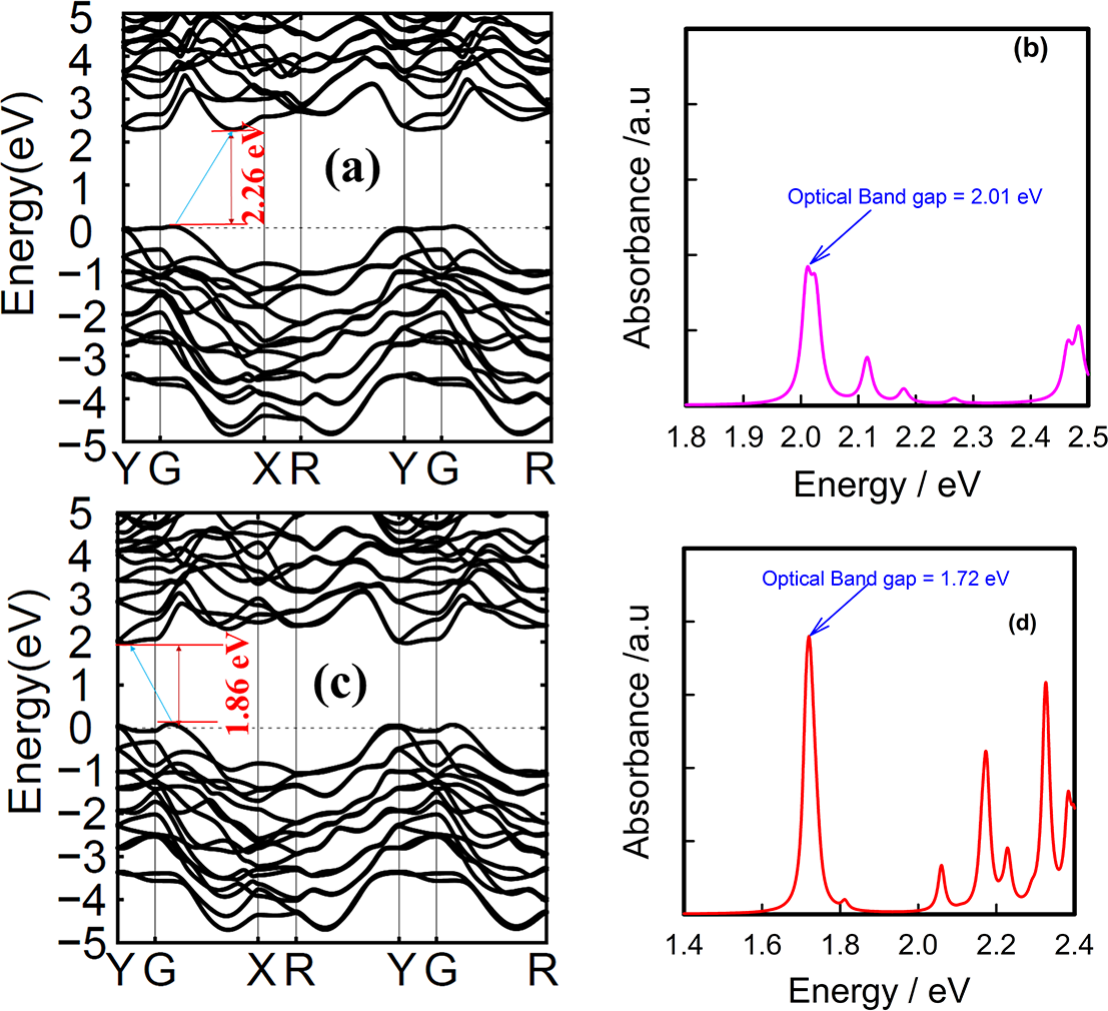
(a,c) GW-band structures and (b,d) BSE-optical absorption spectra of SiAs_2_ and GeAs_2_, respectively.

The quasi-particle band gaps of SiAs_2_ and GeAs_2_ monolayer compounds were found to be 2.26 and 1.86 eV, respectively (Figure 5). These values are comparable with the band gaps obtained by using the HSE-Wannier method (1.91 and 1.64 eV for SiAs_2_ and GeAs_2_, respectively) with similar and negligible variation amongst the two methods. The corresponding first absorption peaks in the absorption spectra are the optical band gaps at 2.01 and 1.72 eV, respectively. Thus the calculated exciton binding energies [43–45] are 0.25 and 0.14 eV for SiAs_2_ and GeAs_2_, respectively. Semiconductors with exciton energies in this range of a few hundred millielectronvolts are supposed to play a key role in photovoltaic applications [46].

## Conclusion

We have presented 2D monolayer compounds of SiAs_2_ and GeAs_2_ as promising light harvesting semiconductor materials for solar cell applications. The extraction of a monolayer of these materials is likely to be feasible by mechanical exfoliation. Moreover, the calculated phonon spectrum reveals its high dynamical stability for both materials. Additionally, the exciton binding energies are quite low and are comparable to quantum dot semiconductors. It might be possible that these semiconductors could be synthesized as quantum dots and studied in further detail. Band gap tuning appears also possible and could be used to tailor the compounds for various electronic applications.

## Supporting Information

Supporting Information shows the band gap variation as a function of tensile strain.

File 1 Additional computational data.
