# Supplementary material for: Computational exploration of two-dimensional silicon diarsenide and germanium arsenide for photovoltaic applications
Source: Beilstein J Nanotechnol. 2018 Apr 19;9:1247–53. doi: 10.3762/bjnano.9.116 (PMC5942365; doi:10.3762/bjnano.9.116)
Supplement: File 1 — Additional computational data. [file Beilstein_J_Nanotechnol-09-1247-s001.pdf]

Supporting Information

for

Computational exploration of two-dimensional silicon diarsenide and germanium arsenide for photovoltaic applications

Sri Kasi Matta, Chunmei Zhang, Yalong Jiao, Anthony O'Mullane and Aijun Du*

Address: School of Chemistry, Physics and Mechanical Engineering, Queensland University of Technology, Garden Point Campus, QLD 4001, Brisbane, Australia

Email: Aijun Du - aijun.du@qut.edu.au

* Corresponding author

Additional computational data

We studied the variation of the band gap as a function of the tensile strain by using the PBE functional (Figure S1). It can be seen that the band gap variation depends on the viewing direction along the lattice. External strain on semiconductor nanostructures influences electronic properties and, thereby, optical properties [1,2].

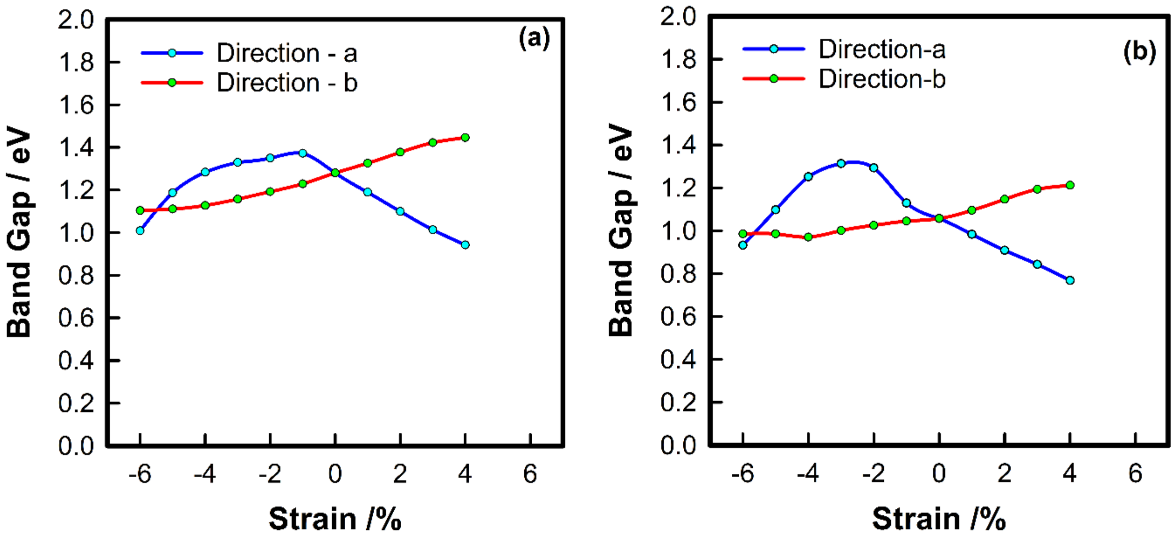

Figure S1: Band gap as a function of biaxial strain calculated with the PBE functional for monolayers of (a) $SiAs_2$ and (b) $GeAs_2$.

References

1. Zhou, M.; Duan, W.; Chen, Y.; Du, A., *Nanoscale* **2015,** *7*, 15168-15174. doi:10.1039/C5NR04431F

2. Ma, F.; Zhou, M.; Jiao, Y.; Gao, G.; Gu, Y.; Bilic, A.; Chen, Z.; Du, A., *Sci. Rep.* **2015,** *5,* 17558. doi:10.1038/srep17558
